# Role of Cannabidiol in the Therapeutic Intervention for Substance Use Disorders

**DOI:** 10.3389/fphar.2021.626010

**Published:** 2021-05-20

**Authors:** Francisco Navarrete, María Salud García-Gutiérrez, Ani Gasparyan, Amaya Austrich-Olivares, Jorge Manzanares

**Affiliations:** ^1^ Instituto de Neurociencias, Universidad Miguel Hernández-CSIC, San Juan de Alicante, Spain; ^2^ Red Temática de Investigación Cooperativa en Salud (RETICS), Red de Trastornos Adictivos, Instituto de Salud Carlos III, MICINN and FEDER, Madrid, Spain

**Keywords:** cannabidiol, substance use disorder, alcohol, cocaine, cannabis, psychostimulant, neurobiology

## Abstract

Drug treatments available for the management of substance use disorders (SUD) present multiple limitations in efficacy, lack of approved treatments or alarming relapse rates. These facts hamper the clinical outcome and the quality of life of the patients supporting the importance to develop new pharmacological agents. Lately, several reports suggest that cannabidiol (CBD) presents beneficial effects relevant for the management of neurological disorders such as epilepsy, multiple sclerosis, Parkinson’s, or Alzheimer’s diseases. Furthermore, there is a large body of evidence pointing out that CBD improves cognition, neurogenesis and presents anxiolytic, antidepressant, antipsychotic, and neuroprotective effects suggesting potential usefulness for the treatment of neuropsychiatric diseases and SUD. Here we review preclinical and clinical reports regarding the effects of CBD on the regulation of the reinforcing, motivational and withdrawal-related effects of different drugs of abuse such as alcohol, opioids (morphine, heroin), cannabinoids, nicotine, and psychostimulants (cocaine, amphetamine). Furthermore, a special section of the review is focused on the neurobiological mechanisms that might be underlying the ‘anti-addictive’ action of CBD through the regulation of dopaminergic, opioidergic, serotonergic, and endocannabinoid systems as well as hippocampal neurogenesis. The multimodal pharmacological profile described for CBD and the specific regulation of addictive behavior-related targets explains, at least in part, its therapeutic effects on the regulation of the reinforcing and motivational properties of different drugs of abuse. Moreover, the remarkable safety profile of CBD, its lack of reinforcing properties and the existence of approved medications containing this compound (Sativex®, Epidiolex®) increased the number of studies suggesting the potential of CBD as a therapeutic intervention for SUD. The rising number of publications with substantial results on the valuable therapeutic innovation of CBD for treating SUD, the undeniable need of new therapeutic agents to improve the clinical outcome of patients with SUD, and the upcoming clinical trials involving CBD endorse the relevance of this review.

## Introduction

Substance Use Disorders (SUD) are chronic and relapsing clinical conditions meeting the diagnostic criteria for drug dependence defined by the Diagnostic and Statistical Manual of Mental Disorders (DSM-5) (APA, 2013) and the World Health Organization’s International Classification of Diseases (ICD-11) (World Health Organization, 2018). SUD are one of the most important health problems globally. In 2017, it was estimated that over 30 million individuals present an SUD leading to more than 31 thousand years lived with disability (YLDs) with a worrying increase (16.7%) over the previous decade (GBD 2017 Disease and Injury Incidence and Prevalence Collaborators, 2018). Furthermore, substance use was indirectly and directly responsible for 11.8 million deaths which implies one in five deaths worldwide (GBD 2017 Disease and Injury Incidence and Prevalence Collaborators, 2018).

Despite the range of the psychosocial and pharmacological therapeutic approaches for substance use treatment, relapse prevalence into drug consumption is estimated between 40 and 75% (Sinha, 2011; Pasareanu et al., 2016; Andersson et al., 2019). This high rate of recurrence is largely due to the ineffectiveness of the available drugs or the lack of specific treatments (e.g., cannabis, cocaine, or amphetamine-type use disorders). Thus, there is a growing need to significantly improve our knowledge about the underlying mechanisms involved in the development of drug dependence to finally design new pharmacological tools with higher efficacy and safety. In this sense, the manipulation of the endocannabinoid system (ECS) by administering cannabinoid compounds has raised much interest due to its close functional involvement in the regulation of emotion, cognition, and reward (Solinas et al., 2008; Marco et al., 2011; Campolongo and Trezza, 2012; Marco and Laviola, 2012; Manzanares et al., 2018; Navarrete et al., 2020).

*Cannabis sativa* plant contains numerous chemical entities including cannabinoids, terpenes, and phenolic compounds (Andre et al., 2016). To date, over 120 cannabinoids have been isolated from the plant (Morales et al., 2017). From these, delta-9-tetrahydrocannabinol (THC) is the main psychotomimetic or hallucinogenic component and the first cannabinoid to be identified and studied. First described and synthesized by Roger Adams in 1942 (Adams, 1942), and then isolated for the first time by Gaoni and Mechoulam in 1964 (Gaoni and Mechoulam, 1964), THC mediates the rewarding properties of cannabis (Zhang et al., 2004). Along with THC, cannabidiol (CBD) is the other most abundant phytocannabinoid in the *Cannabis sativa* plant. It was first synthesized by Roger Adams (Adams, 1942) and isolated by Mechoulam and Shvo in 1963 (Mechoulam et al., 1963), from which a growing interest in its pharmacological actions began to emerge. The results from basic and clinical studies suggested that CBD may present beneficial effects for the management of neurological disorders such as epilepsy (Carlini and Cunha, 1981; Devinsky et al., 2014; Devinsky et al., 2016), multiple sclerosis (Kozela et al., 2011; Giacoppo et al., 2015; Jones and Vlachou, 2020), Parkinson’s (Zuardi et al., 2009; Chagas et al., 2014) or Alzheimer’s diseases (Martín-Moreno et al., 2011; Cheng et al., 2014). Moreover, there is a growing body of evidence suggesting that CBD improves cognition (Osborne et al., 2016) and neurogenesis (Liput et al., 2013; Schiavon et al., 2016), and presents antipsychotic (Zuardi et al., 1991; Moreira and Guimarães, 2005; Long et al., 2006; Leweke et al., 2012; Leweke et al., 2016; Peres et al., 2016), anxiolytic (Guimarães et al., 1990; Moreira et al., 2006; Resstel et al., 2006; Blessing et al., 2015) and antidepressant-like effects (Zanelati et al., 2010; Linge et al., 2016; Sartim et al., 2016). All these potential therapeutic actions of CBD are due to its multiple pharmacological mechanisms. CBD was proposed to directly or indirectly modulate the function of more than 65 targets in the central nervous system (CNS) (Ibeas Bih et al., 2015), including cannabinoid receptors (CB1, CB2), GPR55 receptor, vanilloid receptor TRPV1, serotonin 5HT1a receptor (Bisogno et al., 2001; Russo et al., 2005; Ryberg et al., 2007; Thomas et al., 2007; Campos et al., 2012), the anandamide (AEA) hydrolyzing enzyme (fatty acid amide hydrolase, FAAH) or the adenosine transporter (Carrier et al., 2006; Massi et al., 2008). However, additional studies are needed to precisely determine the target engagement profile of CBD.

Importantly, CBD lacks addictive potential in contrast to THC. Several studies in animals and humans demonstrated the absence of rewarding properties (Parker et al., 2004; Katsidoni et al., 2013; Babalonis et al., 2017; Schoedel et al., 2018). Indeed, recent studies carried out in mice in our laboratory further demonstrate that CBD is not an addictive substance. A range of CBD doses were evaluated in different animal models of addiction commonly used to assess the reinforcing and motivational properties of drugs (conditioned place preference (CPP) and oral self-administration (SA)). Also, withdrawal-related signs were analyzed after the abrupt cessation of CBD chronic administration. Interestingly, CBD did not induce CPP, oral SA or withdrawal-related signs, findings that suggested the lack of rewarding effects of CBD (Viudez-Martínez et al., 2019). Moreover, CBD presents an excellent safety profile supported by both animal and clinical studies (Bergamaschi et al., 2011; Iffland and Grotenhermen, 2017; Taylor et al., 2018). Proof of this is the recent marketing of the drug Epidiolex®, a 99% pure oral CBD extract for the treatment of refractory childhood epilepsies (Lennox-Gastaut and Dravet syndrome) (Sekar and Pack, 2019; Raucci et al., 2020). Likewise, nabiximols is another marketed formulation containing CBD and THC (25 and 27 mg/ml, respectively) under the trade name Sativex®. Nabiximols is an oromucosal spray widely employed for the treatment of muscle spasticity in multiple sclerosis patients (Patti et al., 2016; Giacoppo et al., 2017).

Therefore, the versatile pharmacological profile and safety of CBD support its therapeutic potential in the management of SUD. This review focuses on collecting all the available evidence about the effects of CBD on the different aspects that accompany drug dependence (reinforcement, motivation, contextual conditioning, relapse, withdrawal syndrome or motor sensitization). Also, it covers all the mechanisms proposed to mediate the CBD actions on drug addiction.

## Methods

The literature review consisted of an exhaustive search for scientific information in the Medline database (PubMed). A total of 7 search boxes were employed according to the total of drugs included in the review: cannabis, alcohol, morphine, heroin, amphetamine/methamphetamine, cocaine, and nicotine. These terms were combined with the term “cannabidiol” by the Boolean operator “AND”. All the results for each search were critically analyzed by all the authors to decide the selection of each reference according to the adequacy of its content with the subject matter of the study. No PubMed filters were applied to maximize the selection of all the available and appropriate information. All original articles, systematic reviews or meta-analyses focusing on the effects of CBD on drug addiction were accepted. Those articles not related to the topic of interest, not written in English or to which access was not possible were discarded. In addition, the same searches were performed on the ClinicalTrials.gov database to retrieve all the ongoing clinical studies.

## CBD as a Potential New Pharmacological Tool for the Treatment of SUD

This section details all the available evidence, both pre-clinical and clinical, about the therapeutic potential of CBD in the management of various SUD.

### CBD and Cannabis

Cannabis is the second smoked substance of abuse after tobacco (Hasin et al., 2016) and the most consumed illicit drug worldwide (World Drug Report, 2020). The use of cannabis is growing due to the increasing legalization trend for medicinal and recreational purposes. Furthermore, according to recent studies, THC concentrations in cannabis doubled in the past decade and consequently the content of CBD substantially dropped to an almost non-detectable level (Chandra et al., 2019; Freeman et al., 2019). This scenario facilitates cannabis consumption and may lead to the development of dependence criteria in the context of cannabis use disorder (CUD), affecting approximately 22 million people (Degenhardt et al., 2018). CUD is associated with disruptions in social, occupational, recreational activities and mental health problems. The latter includes impaired cognition abilities and motor coordination, euphoria, depression, psychosis, dependence and withdrawal syndrome (Patel and Marwaha, 2020). Although not medically serious, cannabis withdrawal should be a focus of treatment because one-half of the patients in treatment for CUD report withdrawal-related symptoms and it may serve as a negative reinforcement for relapse to cannabis use in individuals trying to abstain (Budney and Hughes, 2006; Levin et al., 2010; Gorelick et al., 2012).

Nowadays there is no official drug approved for the treatment of CUD by the main drug regulatory authorities (i.e., European Medicine Agency (EMA) or US Food and Drug Administration (FDA)). Many studies were carried out to find out new pharmacotherapies with two main aims: 1) to identify medications to attenuate symptoms of cannabis withdrawal, and 2) to identify medications to reduce subjective and reinforcing effects of cannabis. Some off-label pharmacological interventions targeting distinct neurotransmitter systems involved in drug dependence were investigated (for a recent review see (Sabioni and Le Foll, 2019; Brezing and Levin, 2018)). Recently, the pharmacological modulation of the cannabinoid system gained great interest as a potential therapeutic approach for CUD. Particularly, in the last years CBD attracted much attention as a pharmacological tool for the treatment of CUD due to its safety and multimodal pharmacological profile (García-Gutiérrez et al., 2018) (Table 1). Also, it has been proposed that CBD may reduce the negative psychotropic effects of THC (for a recent review see (Niesink and van Laar, 2013; Freeman et al., 2019)) and might potentiate its positive therapeutic actions (Russo and Guy, 2006; McPartland and Russo, 2014).

**TABLE 1:** Main findings from human and animal studies aimed to evaluate the therapeutic potential of CBD for the treatment of cannabis use disorder.

CBD and cannabis					
Treatment	Doses, route of administration, and treatment duration	Study design/model	Subjects, samples, and gender	Main outcomes	References
Clinical studies					
Nabiximols (CBD:THC)	80 mg CBD:84.6 mg THC/day (maximum daily doses), oromucosal spray, 6 days	2-Site, inpatient, double-blind RCT	Cannabis-dependent treatment seekers *N* = 51 (39 M and 12 F)	↓ CWS	Allsop et al. (2014)
				= Weekly cannabis use	
				↑ withdrawal treatment retention	
Sativex (CBD:THC)	100 mg CBD:108 mg THC/day (maximum daily doses), oromucosal spray, 8 weeks	Double-blind placebo-controlled trial	Community-recruited cannabis dependent patients *N* = 9 (8 M and 1 F)	↓ CWS	Trigo et al. (2016)
				= Craving	
Sativex (CBD:THC) + MET/CBT	105 mg CBD:113,4 mg THC/day (maximum daily doses), oromucosal spray, 12 weeks	Open-label trial	Treatment-seeking community-recruited cannabis-dependent patients *N* = 4 (2 M and 2 F)	↓ cannabis use	Trigo et al. (2016)
				= CWS	
Nabiximols (CBD/THC)	100 mg CBD:108 mg THC/day, oromucosal spray, 8 weeks	Double-blind RCT	Treatment-seeking cannabis-dependent patients *N* = 40 (29 M and 11 F)	↓ cannabis use	Trigo et al. (2018)
				↓ craving	
				= CWS	
Nabiximols (CBD/THC) + CBT	80 mg CBD:86,4 mg THC/day (maximum daily doses), oromucosal spray, 12 weeks	Multi-site, outpatient, double-blind RCT	Treatment-seeking cannabis-dependent patients *N* = 128 (98 M and 30 F)	↓ cannabis use	Bhardwaj et al. (2018); Lintzeris et al. (2019)
				= Craving	
				= CWS	
CBD	300–600 mg/day, capsules, p.o., 11 days	Case report	19 years-old F with cannabis dependence	↓ CWS	Crippa et al. (2013)
				↓ frequency of relapse	
CBD	18–24 mg/day, oromucosal spray, 5 months	Case report	27 years-old M with bipolar disorder and cannabis dependence	↓ anxiety levels	Shannon & Opila-Lehman (2015)
				↓ sleep disturbances	
				Cessation of cannabis use	
CBD	0, 200, 400, 800 mg/day, capsules, p.o., 8 outpatient sessions	Multi-site, double-blind, within-subject RCT	Non-treatment seeking healthy cannabis users *N* = 31 (17 M and 14 F)	= Cannabis self-administration	Haney et al. (2016)
				= Subjective effects	
				= Cannabis ratings	
CBD	0, 200, 400, 800 mg/day, capsules, p.o., 4 weeks	Phase 2a, double-blind RCT	Participants meeting CUD criteria *N* = 82 (59 M and 23 F)	↓ cannabis use	Freeman et al. (2020)
				↓ urinary THC-COOH:creatinine ratio	
CBD	200 mg/day, capsules, p.o., 10 weeks	Open-label trial	Regular cannabis users *N* = 18 (14 M and 4 F)	↓ cannabis-induced hippocampal disturbances	Beale et al. (2018)
CBD	200 mg/day, capsules, p.o., 10 weeks	Open-label trial	Regular cannabis users *N* = 16 (M)	↓ cannabis-induced euphoria	Solowij et al. (2018)
				↓ depressive and psychotic-like symptoms	
				↑ attentional switching, verbal learning, and memory	
Epidiolex (CBD)	800 mg/day (maximum daily dose), solution, p.o., 6 weeks	Double-blind RCT	Cannabis-dependent patients *N* = 10 (4 M and 6 F)	↑ cannabis use	ClinicalTrials.gov ID:NCT03102918
CBD	300–600 mg/day, capsules, p.o., 6 weeks	Double-blind RCT	Patients with psychosis and cannabis abuse *N* = 130 (M/F)	- Cannabis cessation	ClinicalTrials.gov ID: NCT04105231
				- Psychotic symptoms (no results posted yet)	
CBD	600 mg/day, p.o., 12 weeks	Double-blind RCT	Regular cannabis users with recent-onset psychosis *N* = 84 (M/F)	- Change in BPRS score	ClinicalTrials.gov ID: NCT03883360
				- Change in MATRICS score	
				- Change in serum [THC-COOH] (no results posted yet)	
Animal studies					
CBD	5, 10, 30 mg/kg, i.p., acute treatment	Spontaneous cannabinoid withdrawal	C57BL/6J mice *N* = 180 (M)	↓ anxiety level	Navarrete et al. (2018)
				↓ hyperactivity	
				↓ withdrawal somatic signs	
CBD	0–20 mg/kg, i.p., chronic treatment	Precipitated cannabinoid withdrawal	C57BL/6J mice *N* = 335 (M)	= Withdrawal somatic signs	Myers et al. (2019)
				↓ anxiety level	

CBD, cannabidiol; THC, tetrahydrocannabinol; RCT, randomized clinical trial; CWS, cannabis withdrawal syndrome; MET, motivational enhancement therapy; CBT, cognitive behavioral therapy; BPRS, Brief Psychiatric Rating Scale; MATRICS, MATRICS Consensus Cognitive Battery; CUD, cannabis use disorder; M, male; F, female; p.o., per os (oral administration); i.p., intraperitoneal injection; ↑, increase; ↓, decrease; = , no effect.

Several studies carried out with cannabis users classified them according to the higher or lower CBD:THC ratio of their smoked cannabis. Interestingly, CBD reduces the rewarding effects of THC since cannabis smokers (*n* = 94) with high CBD:THC ratio showed reduced attentional bias to drug stimuli and lower self-rated liking of cannabis (Morgan et al., 2010). Another study recruited cannabis users (*n* = 134) that were classified based on levels of CBD in their own chosen cannabis, low (0.14%) vs. high (0.75%). CBD-enriched cannabis did not cause the deficits of immediate and delayed prose recall that were caused by CBD-poor cannabis (Morgan et al., 2010), and users habitually exposed to CBD-rich cannabis relatively preserved recognition memory vs. CBD-poor cannabis users (Morgan et al., 2012). Likewise, the analysis of cannabinoids in hair samples collected from 140 individuals allowed the comparison between “THC only”, “THC + CBD” and “no cannabinoid” groups in terms of schizophrenia-like symptoms. The “THC + CBD” group showed lower levels of positive psychotic symptoms compared with the “THC only” and “no cannabinoid” groups (Morgan and Curran, 2008). These findings are relevant for the therapeutic and public health implications, suggesting that for recreational cannabis users and for those patients taking medicinal cannabis, a more balanced CBD to THC concentration would improve therapeutic endpoints while minimizing side effects.

In a recent clinical trial with healthy volunteers (*n* = 17) experienced with cannabis (not regular users), functional Magnetic Resonance Imaging (fMRI) studies were performed to investigate the effects of THC (8 mg) and THC + CBD (8 mg + 10 mg) on resting-state brain functional connectivity. CBD restored the THC-induced disruption of the salience network, effect that authors associated with its potential to treat disorders of salience such as psychosis and addiction (Wall et al., 2019). Likewise, another study enrolling frequent and infrequent cannabis users (*n* = 36) evaluated the effects of THC alone (8 mg) and THC combined with low (4 mg) or high (400 mg) doses of CBD. The results showed that only the high dose of CBD reduced the intoxicating effects of THC (Solowij et al., 2019). In addition, the cannabinoid spray Sativex (1:1 ratio of CBD:THC) at low doses reduces some of the effects produced by THC, including subjective ratings of intoxication and abuse/dependence (Robson, 2011; Schoedel et al., 2011). Also, CBD:THC (1:1 or 1:10 ratios) reversed the conditioned place aversion (CPA) induced by the acute injection of THC (10 mg/kg) in Long Evans rats (Vann et al., 2008).

The protective effects of CBD alone on THC-induced impairments were extensively explored in preclinical and clinical studies. For instance, the administration of CBD (0.5 mg/kg) to rhesus monkeys challenged with THC (0.2, 0.5 mg/kg) significantly attenuated THC-induced cognitive disturbances (Wright Jr. et al., 2013). CBD reduced anxiety and improved fear-related responses induced by THC in male Sprague Dawley rats via a bidirectional control of ERK1-2 phosphorylation (Hudson et al., 2019). In C57BL/6J mice, CBD (3 mg/kg) significantly blunted the cognitive alterations induced by THC (1 mg/kg) administration in an object recognition task (Aso et al., 2019). In the clinical setting, CBD (1 mg/kg) blocked the anxiety induced by THC (0.5 mg/kg) (Zuardi et al., 1982). Furthermore, CBD pre-treatment (600 mg) inhibited THC (1.5 mg)-induced paranoia, inhibited the detrimental effects of THC on episodic memory and decreased the proportion of participants experiencing clinically significant acute THC psychosis (Englund et al., 2013). Importantly, the restorative properties of CBD were also explored in 18 regular cannabis users (heavy and light users) enrolled in a 10 weeks open-label pragmatic trial. Authors measured baseline and post-CBD hippocampal subregions volumes by structural fMRI. CBD restored cannabis-induced anatomical disturbances in the subicular and CA1 subfields of the hippocampus (HIPP) in current cannabis users, especially in those with greater lifetime exposure (Beale et al., 2018). In the same study, CBD improved psychological symptoms (depressive and psychotic-like traits) and cognition (attentional switching, verbal learning, and memory) in dependent cannabis users (Solowij et al., 2018).

Considering the significant CBD-mediated attenuation of the negative outcomes induced by THC, as well as the promising effects of cannabinoid agonist substitution approaches employing synthetic derivatives of THC (e.g., dronabinol, nabilone) (Haney et al., 2004; Budney et al., 2007; Haney et al., 2008; Haney et al., 2013; Vandrey et al., 2013), there has been a growing interest in the therapeutic potential of the combination CBD:THC for the treatment of distinct aspects of CUD (Allsop et al., 2015). Cannabis-dependent treatment seekers (*n* = 51) received nabiximols (maximum daily doses: 80 mg CBD/86.4 mg THC, oromucosal spray) or placebo with standardized psychosocial interventions. Nabiximols significantly reduced the severity of cannabis withdrawal and prolonged the retention in withdrawal treatment (Allsop et al., 2014). Later, Trigo et al. first explored the effects of fixed or self-titrated dosages of Sativex (maximum daily doses: 100 mg CBD:108 mg THC, oromucosal spray) on cannabis withdrawal and craving. High fixed Sativex doses were well tolerated and significantly attenuated cannabis withdrawal while craving was similar compared to placebo (Trigo et al., 2016). Second, the effects of self-titrated Sativex doses combined with motivational enhancement therapy and cognitive behavioral therapy (MET/CBT) on cannabis withdrawal, use and craving were evaluated. Self-titrated Sativex (maximum daily doses: 105 mg CBD/113.4 mg of THC, oromucosal spray) with MET/CBT significantly decreased cannabis use and prevented cannabis withdrawal under abstinence conditions in these case series (Trigo et al., 2016). Third, the same previous experimental design was employed to evaluate the tolerability, safety, and efficacy of nabiximols (maximum daily doses: 100 mg CBD:108 mg THC, oromucosal spray). Cannabis use as well as craving were reduced in nabiximols-treated patients compared with placebo, although no differences were found on withdrawal scores (Trigo et al., 2018). Finally, a clinical trial examined the safety and efficacy of nabiximols treatment (up to 32 oromucosal sprays containing 86.4 mg THC/80 mg CBD), combined with individual CBT (Bhardwaj et al., 2018). Interestingly, the nabiximols group reported significantly less days using cannabis than the placebo group while both groups improved to a comparable degree on a range of secondary cannabis-related and general health and psychosocial outcomes (Lintzeris et al., 2019).

One of the major concerns of the cannabinoid replacement therapy is whether the presence of THC in nabiximols could be problematic, especially in the still unexplored long-term treatment of CUD. For this reason, special attention has been paid to the evaluation of the clinical efficacy of CBD alone. The potential therapeutic usefulness of CBD for the treatment of CUD was investigated in some case report clinical studies. Crippa et al. administered CBD for 11 days (300 mg on day 1, 600 mg on days 2–10, and 300 mg on day 11, capsules, p.o.) to a 19 year-old female with cannabis dependence who experienced withdrawal syndrome when she tried to cease cannabis use. Daily assessments showed a rapid decrease in withdrawal symptoms leading to a score of zero in all tests by day 6. A 6 months follow-up showed a relapse in cannabis use but at a lower frequency (once or twice a week vs. 7 days a week) (Crippa et al., 2013). Another case report study evaluated the use of a CBD oil in a 27 year-old male presenting a long-standing diagnosis of bipolar disorder and a daily addiction to cannabis use. After initiating the treatment with CBD oil (18–24 mg/day, oromucosal spray), the patient reported a decrease in the anxiety level and sleep disturbances, as well as a complete cessation of cannabis use (Shannon and Opila-Lehman, 2015). A multi-site clinical study analyzed the effects of oral CBD (0, 200, 400, 800 mg, capsules, p.o.) on the reinforcing, subjective, cognitive, and physiological effects of smoked cannabis. CBD was administered 90 min prior to smoking half of a cannabis cigarette by non-treatment-seeking healthy cannabis users (*n* = 31) during 8 outpatient sessions. No difference was found in comparison with placebo-treated patients (Haney et al., 2016). This may be due to the acute CBD treatment, the study population (non-treatment-seeking patients) or the poor bioavailability of oral CBD. Recently, a phase 2a, double-blind, placebo-controlled, randomized clinical trial was carried out to identify efficacious doses of CBD (200, 400 and 800 mg, capsules, p.o., 4 weeks) for the treatment of CUD. Following a 2-stages design with 82 participants meeting CUD criteria from DSM-5 (48 in stage 1 and 34 in stage 2), CBD efficacy was determined according to urinary 11-nor-9-carboxy-δ-9-tetrahydrocannabinol (THC-COOH):creatinine ratio and/or increased days per week with abstinence from cannabis during treatment. CBD 400 and 800 mg doses were well tolerated and more efficacious than placebo at reducing cannabis use (Freeman et al., 2020). Another recent clinical study also explored the effects of CBD (Epidiolex, up to 800 mg, solution, p.o., 6 weeks treatment period) in cannabis dependent subjects (*n* = 10). Although no significant differences were found, cannabis consumption was higher in the CBD-treated group. However, as stated by the authors, more participants are necessary to draw definitive conclusions from this study (Clinicaltrials.gov identifier: NCT03102918). Interestingly, two clinical trials have been recently registered (Clinicaltrials.gov identifiers: NCT04105231 and NCT03883360) to explore the effects of long-term administration of CBD (up to 600 mg, capsules, p.o., 6 or 12 weeks, respectively) on psychiatric symptoms, cognition, and cannabis consumption in patients with recent-onset psychosis and comorbid cannabis use.

Apart from the valuable information provided by clinical studies, it is essential to analyze the effects of CBD on behavioral and neurobiological alterations related with cannabis dependence at the preclinical level. For that purpose, our laboratory was the first to explore CBD actions (5, 10 and 20 mg/kg, i.p.) in an animal model of spontaneous cannabinoid withdrawal syndrome developed after 7 days of treatment with CP-55,940 (a 45-fold more potent cannabinoid 1 receptor (CB1R) agonist compared to THC) (Aracil-Fernández et al., 2013). Withdrawal-related behavioral signs were evaluated by measuring motor activity, somatic signs, and anxiety-like behavior in abstinent C57BL/6J mice treated with CBD or its corresponding vehicle. In addition, real-time PCR (RT-PCR) analyses were performed to evaluate changes in the gene expression of relevant targets of the cannabinoid, dopaminergic, and opioidergic systems. Interestingly, CBD administration significantly blocked the increase in motor-activity, number of rearings, rubbings, and jumpings associated with spontaneous cannabinoid withdrawal, and normalized the decrease in the number of groomings. Furthermore, the anxiogenic-like effect observed in abstinent mice was completely abolished by CBD. These effects were associated with a CBD-induced up-regulation of tyrosine hydroxylase (TH) in the ventral tegmental area (VTA) and cannabinoid 2 receptor (CB2R) in the nucleus accumbens (NAcc), whereas a down-regulation of mu-opioid receptor (MOR) and CB1R in the NAcc (Navarrete et al., 2018). Also, a recent study was aimed to evaluate if CBD (0–20 mg/kg, i.p.) improves cognitive deficits and withdrawal signs induced by cannabinoid CB1/CB2 receptor agonists such as THC. CB1R antagonist (SR141716) administration precipitated withdrawal signs in chronically THC-treated C57BL/6J mice and they were not attenuated by CBD. However, the lack of CBD-induced withdrawal signs or cognitive performance impairment, together with the robust anxiolytic effect led the authors to conclude that CBD as a monotherapy might be a safer pharmacological agent for the treatment of several disorders (Myers et al., 2019).

According to the previous evidence, it seems that CBD could play a crucial role in the management of CUD. The clinical studies that are underway as well as future investigations will be decisive to determine the therapeutic application of CBD to treat cannabis addiction.

### CBD and Alcohol

Problematic alcohol use is an important risk factor for many health problems significantly contributing to the global burden of disease (Collaborators, 2018). In 2016, harmful alcohol use caused 3 million of deaths worldwide and 132.6 million disability-adjusted life years (DALYs) (OMS, 2019). Alcohol Use Disorder (AUD) is one of the most common addictive disorders with a greatest health and socioeconomic impact. The prevalence of AUD varies from 13 to 30% in most western countries (Grant et al., 2015; WHO, 2018). Current options for AUD treatment are scarce and have limited efficacy. To date, there are only four drug-based treatments approved for AUD by the FDA and EMA: naltrexone, nalmefene, acamprosate, and disulfiram (Soyka and Müller, 2017). Despite the optimization of pharmacological and psychosocial interventions for the management of AUD, at least 60% of alcoholic patients usually relapse during the first 6 months after dishabituation treatment (Maisto et al., 2006; Kirshenbaum et al., 2009; Witkiewitz, 2011; Durazzo and Meyerhoff, 2017). Thus, the need for new pharmacological approaches proving higher efficacy in alcohol relapse avoidance and maintenance of abstinence is evident. In this sense, CBD has recently attracted attention because of its ability to modulate the reinforcing and motivational effects of alcohol, as well as to improve the damage produced by alcohol in the liver or CNS (De Ternay et al., 2019; Turna et al., 2019) (Table 2).

**TABLE 2:** Main findings from clinical and animal studies aimed to evaluate the therapeutic potential of CBD for the treatment of alcohol use disorder.

CBD and alcohol					
Treatment	Doses, route of administration, and treatment duration	Study design/model	Subjects, samples, and gender	Main outcomes	References
Clinical studies					
CBD	600 and 1,200 mg/day, p.o., 4 + 4 weeks	Double-blind RCT	Patients with moderate or severe AUD (DSM-5) *N* = 40 (M/F)	- TLFB assessment of alcohol consumption in serum	ClinicalTrials.gov ID: NCT03252756
				- Change in % CDT assessment of alcohol consumption in serum (no results posted yet)	
CBD	600 mg/day, p.o., 6 weeks	Double-blind RCT	Patients with AUD and PTSD comorbidity *N* = 48 (M/F)	- Number of drinks per day with TLFB (no results posted yet)	ClinicalTrials.gov ID: NCT03248167
CBD	800–1,200 mg/day, capsules, p.o., 4 days	Double-blind RCT	Patients with AUD undergoing alcohol withdrawal *N* = 52 (M/F)	- diazepam use over the 5 days withdrawal period (no results posted yet)	ClinicalTrials.gov ID: NCT04205682
Animal studies					
CBD	30, 60, 120 mg/kg, i.p., 30 mg/kg/day, s.c. (continuous controlled release), chronic treatment	VC, ESA	C57BL/6J mice *N* = 40 (M)	↓ ethanol intake and preference	Viudez-Martínez et al. (2018a)
				↓ motivation to ethanol consumption	
				↓ ethanol relapse	
CBD	15 mg/kg/day, t.d., 7 days	ESA, DRT	Wistar rats *N* = 52 (M)	↓ context-induced and stress-induced reinstatement	Gonzalez-Cuevas et al. (2018)
				↓ impulsivity level in rats with alcohol dependence history	
CBD	2.5 mg/kg CBD ±2.5 mg THC, i.p., acute treatment	Ethanol-induced locomotor sensitization	DBA/2 mice *N* = 84 (M)	↓ motor sensitization to ethanol	Filev et al. (2017)
CBD + THC					
CBD ± NTX	20 mg/kg/day CBD, s.c. (continuous controlled release) ± 0.7 mg/kg NTX; p.o., 0.3 mg/kg WAY, i.p., chronic treatment	ESA	C57BL/6J mice *N* = 140 (M)	↓ motivation to ethanol consumption (CBD + NTX) → abolished by WAY	Viudez-Martínez et al. (2018b)
WAY					
CBD	15, 30, 60, 90 mg/kg, i.p., chronic treatment	Binge drinking	C57BL/6J mice *N* = 120 (60 M and 60 F)	↓ ethanol intake (30, 60 and 90 mg/kg, repeated administration, M)	Viudez-Martínez et al. (2020)
				↓ ethanol intake (90 mg/kg, acute and repeated administration, F)	
CBD	20, 40 mg/kg, i.p., repeated treatment	Binge ethanol exposure	Sprague-dawley rats (M)	↓ ethanol-induced hippocampal and entorhinal cortical neurodegeneration	Hamelink et al. (2005)
CBD	1, 1.0, 2.5 and 5.0% gel, t.d., 40 mg/kg, i.p., repeated treatment	Binge ethanol exposure	Sprague-dawley rats (M)	↓ FJB + cells in the entorhinal cortex	Liput et al. (2013)
CBD	5 mg/kg, i.p., 5 days	Binge ethanol exposure	C57BL/6J mice (M)	↑ alcohol-induced liver steatosis	Yang et al. (2014)
				↓ alcohol-mediated oxidative stress	
				↓ JNK MAPK activation	
				↑ autophagy	
CBD	5, 10 mg/kg, i.p., 11 days	Chronic ethanol exposure	C57BL/6J mice (M)	↓ alcohol feeding-induced serum transaminase elevations	Wang et al. (2017)
				↓ hepatic inflammation	
				↓ oxidative/nitrative stress	

CBD, cannabidiol; THC, tetrahydrocannabinol; RCT, randomized clinical trial; AUD, alcohol use disorder; DSM-5, Diagnostic and Statistical Manual of Mental Disorders; TLFB, Time-line Follow-back scale; CDT, carbohydrate deficient transferrin; VC, voluntary consumption; ESA, ethanol self-administration; DRT, delayed reinforcement task; WAY, WAY-100635 (5HT1a selective antagonist); M, male; F, female; p.o., per os (oral administration); i.p., intraperitoneal injection; t.d., transdermal; ↑, increase; ↓, decrease; = , no effect.

Our laboratory was the first to publish relevant data regarding the effects of CBD on ethanol reinforcement, motivation, and relapse in C57BL/6J male mice. Voluntary ethanol consumption (VEC) and oral ethanol SA procedures were employed. First, VEC was evaluated in a two-bottle choice paradigm in which mice were repeatedly administered with different doses of CBD (30, 60 and 120 mg/kg, i.p.). Ethanol consumption and preference were significantly reduced by CBD in a dose-dependent manner. Second, oral ethanol SA was carried out in operant skinner boxes to evaluate the effects of a single administration of CBD in a microparticle formulation providing a constant release (30 mg/kg/day, s.c.). Interestingly, CBD significantly reduced the number of active lever presses and ethanol intake under fixed-ratio 1 (FR1) and fixed-ratio 3 (FR3) schedules, as well as the breaking point that measures the motivation to drink alcohol. Third, the effects of CBD on alcohol relapse were also analyzed in the oral ethanol SA paradigm with some modifications. The administration of CBD (120 mg/kg, i.p.) significantly reduced the number of active lever presses and ethanol intake during relapsing conditions. Importantly, these effects were accompanied by changes on the relative gene expression (RT-PCR) of selected dopaminergic, opioidergic and cannabinoid targets. Briefly, CBD induced a down-regulation of TH in the VTA and MOR, CB1R and G-protein coupled receptor 55 (GPR55) gene expressions in the NAcc whereas CB2R mRNA levels were increased in the NAcc (Viudez-Martínez et al., 2018a). Shortly thereafter, Gonzalez-Cuevas et al. demonstrated that CBD transdermal administration (fast-drying 2.5% hydroalcoholic gel formulation, 15 mg/kg/day, 7 days) significantly attenuated the context- and stress-induced reinstatement for ethanol seeking, and this effect lasted up to 5 months. In addition, CBD fully reversed the high impulsivity level showed by rats with an EtOH dependence history (Gonzalez-Cuevas et al., 2018). On the other hand, the effects of CBD alone or in combination with THC (2.5 mg/kg each, i.p., 4 days) on ethanol-induced locomotor sensitization were also evaluated in DBA/2 mice. THC alone or combined with CBD, but not CBD alone, significantly inhibited the expression of sensitization to ethanol in this paradigm (Filev et al., 2017).

The combination of different drugs is a commonly used procedure for the treatment of AUD. This strategy usually provides a greater effect and prevents certain dose-related side effects by using lower doses of each drug than the ones employed in monotherapy. Taking into consideration this approximation, our group was also aimed to explore whether the combination of CBD with naltrexone (NTX) might reduce alcohol consumption and motivation to drink in C57BL/6J mice to a higher extent. For that purpose, the effects of a sub-effective dose of NTX (0.7 mg/kg, p.o.), CBD (20 mg/kg/day, s.c., microparticles formulation for continuous controlled release for 3 weeks) or their combination were evaluated. Interestingly, the administration of CBD plus NTX was the only treatment able to reduce motivation and ethanol intake in the oral ethanol SA. Also, these effects were associated with a down-regulation in the gene expression of TH in the VTA, MOR in the NAcc, and serotonin 1a receptor (5HT1a) in the dorsal raphe. To elucidate the role of 5HT1a receptors in the mechanisms that could underlie CBD plus NTX effects on ethanol reinforcement and motivation, the 5HT1a antagonist WAY 100635 was concomitantly administered. Pretreatment with this compound significantly blocked the effects of CBD plus NTX, a finding that supports the involvement of 5HT1a receptors (Viudez-Martínez et al., 2018a).

One of the major concerns of harmful ethanol consumption is the binge drinking pattern that has become a major public health problem in modern societies (Lannoy et al., 2019). Nevertheless, the available pharmacological options for binge drinking management are scarce and limited (Rolland and Naassila, 2017). In this respect, therapeutic usefulness of CBD for the treatment of binge drinking patterns was analyzed also by our group taking into consideration gender differences. The effects of CBD on ethanol binge drinking were explored in male and female C57BL/6J mice by using the drinking in the dark procedure. Repeated CBD administration (15, 30 and 60 mg/kg, i.p.) significantly reduced ethanol intake only in males and was associated with a down-regulation of TH gene expression in the VTA, and MOR and CB1R gene expressions in the NAcc. Interestingly, a higher CBD dose (90 mg/kg, i.p.) significantly reduced ethanol intake under acute and repeated administration patterns not only in males but also in females (Viudez-Martínez et al., 2020). Except for these findings, previous studies provided evidence of CBD neuroprotective actions in rodent models of ethanol binge intoxication. In 2005, Hamelink et al. demonstrated that CBD (40 mg/kg, i.p.) significantly reduced the number of degenerated argyrophilic neurons in the dentate gyrus of the HIPP and the entorhinal cortex of Sprague Dawley rats exposed to a 4 days ethanol binge administration (Hamelink et al., 2005). Also, Liput and cols followed a similar procedure showing a significant CBD-mediated reduction in the neurodegeneration induced by ethanol binge treatment reflected in a lower number of Fluoro-Jade B positive cells in the entorhinal cortex (Liput et al., 2013). Finally, it is worth to mention that CBD might also present protective actions against alcohol-induced liver disease, attenuating hepatic steatosis and metabolic dysregulation by anti-inflammatory and antioxidant mechanisms in animal models of repeated ethanol exposure (Yang et al., 2014; Wang et al., 2017).

Taking into consideration the promising preclinical data pointing out CBD as a potential therapeutic tool for AUD, clinical studies were recently initiated. In 2019, a randomized, double blinded and proof-of-concept clinical trial was started in United States (New York) to assess the effects of extended treatment with CBD (600 and 1,200 mg/day, 4 weeks for each dosing, p.o.) compared to placebo in 40 patients with severe AUD (NCT03252756). In the same year, another randomized, double-blind and placebo-controlled clinical trial began also in United States (New York) to determine whether CBD (600 mg, 6 weeks, p.o.) is effective in treating AUD in individuals (48 participants) with moderate or severe AUD and comorbid posttraumatic stress disorder (PTSD) (NCT03248167). Finally, another randomized, double-blind, and placebo-controlled clinical trial to explore the effectiveness and tolerability of CBD (1,200 mg/day 1, 800 mg/days 2–4, p.o.) in the treatment of alcohol withdrawal symptoms in an inpatient setting (52 participants) in Australia (NCT04205682) is expected to start in 2020.

Thus, the great and growing interest in CBD as a new drug for AUD management is more than evident. However, further studies are warranted to shed light on the underlying brain mechanisms involved as well as on pharmacokinetics aspects such as dose, treatment duration, route of administration or pharmaceutical formulation.

### CBD and Opioids

Opioid use disorder (OUD) could be defined as a chronic, relapsing illness, associated with significantly increased rates of morbidity and mortality. In the United States, 5.1 million people (1.9 percent of persons age 12 or older) were estimated in 2015 to have used heroin at some point in their lives (Hser et al., 2015; Substance Abuse and Mental Health Services Administration, 2016). Patients may develop OUD by acquiring illegal opioid drugs (e.g., heroin), by obtaining them legally but use them for not legitimate medical purposes (morphine, fentanyl, oxycodone, etc.), or at doses in excess to the needed for the medical condition (APA, 2013). One of the major concerns associated with OUD is the occurrence of opioid overdose with high rates of mortality, especially in United States were recent data show a significant increase (Rudd et al., 2016). Currently, the FDA and the EMA authorize the marketing of three classes of medications: 1) the short-acting opioid antagonist naloxone employed to reverse the life-threatening effects of opioid overdose, 2) oral opioid agonists methadone and buprenorphine, highly effective and widely employed in opioid maintenance programs to achieve abstinence and avoid relapse, and 3) the alpha 2-adrenergic agonist lofexidine, recently approved by the FDA representing the first non-opioid medication indicated for mitigation of symptoms associated with acute opioid withdrawal and for facilitation of the completion of opioid discontinuation treatment (Gorodetzky et al., 2017; Guo et al., 2018). Nowadays, buprenorphine (BPN) has been proposed as one of the first-line treatments for OUD management due to its low abuse potential, reduced risk of overdose and flexible dosing in comparison with methadone (Li et al., 2014). However, recent evidence points out poor patient retention in BPN maintenance (Bell, 2014; Hser et al., 2014; Mattick et al., 2014; Burns et al., 2015). This fact together with the limited efficacy of current options for the treatment of OUD motivates the development of new mechanistically based pharmacological strategies that go beyond treating symptoms associated with opioid withdrawal syndrome to relapse. CBD may serve as a new therapeutic strategy for the treatment of OUD, not simply for withdrawal symptomatic relief partly due to its anxiolytic properties, but also to reduce craving and avoiding relapse (Table 3).

**TABLE 3:** Main findings from clinical and animal studies aimed to evaluate the therapeutic potential of CBD for the treatment of opioid use disorder.

CBD and opioids					
Treatment	Doses, route of administration, and treatment duration	Study design/model	Subjects, samples, and gender	Main outcomes	References
Clinical studies					
Epidiolex (CBD)	400 or 800 mg/day, p.o., 3 days	Double-blind RCT	Patients with heroin use disorder *N* = 42 (35 M and 7 F)	↓ craving and anxiety after acute, short term and long-term evaluation	Hurd et al. (2019)
				↓ heart rate after acute and short-term evaluation	
				↓ cortisol levels	
APH-1501 (>98.5% CBD, <0.3 THC)	400, 600, 800 mg/day, capsules, p.o., 28 days	Triple-blind RCT	Opioid-dependent patients *N* = 32 (M/F)	- Incidence of treatment adverse effects	ClinicalTrials.gov ID: NCT03813095
				- Pharmacokinetics of APH-1501 (no results posted yet)	
Epidiolex (CBD)	800 mg/day, oral solution, p.o., 2 days	Open-label	Methadone-maintained participants undergoing spontaneous withdrawal *N* = 50 (M/F)	- Safety as assessed by number of adverse events	ClinicalTrials.gov ID: NCT04238754
				- Number of participants whose AST/ALT levels >3x upper limit of normal	
				- Feasibility of spontaneous withdrawal model as assessed by change in withdrawal scores (no results posted yet)	
Animal studies					
CBD ± THC	10 mg/kg CBD ±2 mg/kg THC, i.p., acute treatment	Naloxone-induced morphine abstinence	Sprague-dawley rats *N* = 33 (M)	↓ morphine withdrawal signs (CBD + THC combination)	Hine et al. (1975)
CBD	5, 10, 20 mg/kg, i.p., acute treatment	Naloxone-induced morphine withdrawal	Swiss-webster mice (M)	**↑** dose of naloxone needed to induce morphine withdrawal jumping in 50% of the animals (ED50)	Bhargava. (1976)
				↓ jumping, defecations, and rearing behaviors	
CBD	5, 20, 80 mg/kg, i.p., acute treatment	Quasi-morphine withdrawal syndrome	Sprague-dawley rats (M)	= Withdrawal score	Chesher & Jackson. (1985)
CBD	5 mg/kg, i.p., acute treatment	Morphine-induced ICSS	Sprague-dawley rats (M)	⊗ reward-facilitating morphine effects → abolished by WAY	Katsidoni et al. (2013)
CBD	5, 10 mg/kg, s.c., acute treatment	Morphine-induced CPP, naltrexone-induced CPA	Wistar rats N = 295 (M)	↓ CPP	de Carvalho & Takahashi. (2017)
				⊗ morphine priming- or stress-induced CPP reinstatement	
				⊗ naltrexone-induced CPA	
CBD	2.5, 5, 10, 20 mg/kg, i.p., acute treatment	Morphine-induced CPP	C57BL/6 mice *N* = 100 (M)	↓ CPP	Markos et al. (2018)
CBD	5, 10, 20 mg/kg, i.p., acute treatment	Heroin-induced ISA	Long-evans rats (M)	= Heroin ISA	Ren et al. (2009)
				= priming-induced heroin seeking	
				↓ cue-induced heroin seeking	

CBD, cannabidiol; THC, tetrahydrocannabinol; RCT, randomized clinical trial; CPP, conditioned place preference; CPA, conditioned place aversion; ISA, intravenous self-administration; WAY, WAY-100635 (5HT1a selective antagonist); M, male; F, female; p.o., per os (oral administration); i.p., intraperitoneal injection; ↑, increase; ↓, decrease; = , no effect; ⊗, blockade.

The first evidence on the possible therapeutic utility of CBD in the regulation of pharmacologically induced morphine withdrawal was reported in 1975. The effects of CBD (10 mg/kg, i.p.), alone or combined with THC (2 mg/kg, i.p.), on naloxone-induced morphine abstinence were evaluated in male Sprague-Dawley rats. THC and especially CBD plus THC combination significantly attenuated morphine withdrawal signs whereas no effects were found with CBD alone (Hine et al., 1975). Shortly another study evaluated the effects of some cannabinoid compounds on naloxone-precipitated abstinence signs in Swiss-Webster male mice. Interestingly, CBD (5 and 10 mg/kg, i.p.) significantly increased the dose of naloxone needed to induce morphine withdrawal jumping in 50% of the animals (ED50), although it was not the most effective cannabinoid (Bhargava, 1976). To further elucidate the therapeutic potential of cannabinoid compounds to modulate morphine withdrawal, Chesher and Jackson analyzed whether cannabinol, CBD or THC attenuate the signs associated with the quasi-morphine withdrawal syndrome in male Sprague-Dawley rats. THC and cannabinol significantly reduced the withdrawal score whereas CBD was without effect at the dosage levels used (5, 20 and 80 mg/kg, i.p.) (Chesher and Jackson, 1985).

More recently, it was reported that CBD might interfere with brain reward mechanisms responsible for the expression of the acute reinforcing properties of opioids such as morphine. Indeed, authors showed that CBD inhibited the reward-facilitating effect of morphine employing the intracranial self-stimulation (ICSS) paradigm. Interestingly, pre-treatment with an intra-dorsal raphe injection of the selective 5HT1a receptor antagonist WAY-100635 reversed the effects of CBD, suggesting the involvement of these receptors in the CBD-mediated inhibition of morphine-induced reward (Katsidoni et al., 2013). Also, the efficacy of CBD to regulate morphine-induced CPP was investigated by two independent studies. First, in male Wistar rats the administration of CBD (10 mg/kg, i.p.) blocked place conditioning behavior and reinstatement induced by a priming dose of morphine or stress exposure (de Carvalho and Takahashi, 2017). Second, in male C57BL/6J mice the same dose of CBD also significantly attenuated morphine-induced CPP (Markos et al., 2018).

Heroin is a morphine derivative with a higher addictive power and is usually consumed first by patients starting the use of opioids (Cicero et al., 2017). To evaluate if the administration of CBD could modify the reinforcing and motivational properties of heroin, Ren et al. employed an animal model of heroin intravenous SA. They assessed the actions of CBD on heroin SA and relapse induced by a heroin prime injection or the exposure to conditioned contextual cues. The administration of CBD (5 or 20 mg/kg, i.p.) was without effect on heroin consumption and did not prevent relapse by a priming dose of heroin. However, it significantly attenuated the reinstatement of cue-induced heroin seeking. Interestingly, CB1R and glutamatergic mGluR5 and GluR1 gene and/or protein alterations were normalized with CBD treatment (Ren et al., 2009). A few years later, a double-blind randomized placebo-controlled clinical trial evaluated the utility of CBD (400 or 800 mg) to reduce cue-induced craving and anxiety in drug-abstinent patients with heroin use disorder. The results showed that the administration of CBD reduces both craving and anxiety induced by the presentation of heroin-related salient drug cues. Furthermore, CBD also attenuated drug cue-induced physiological measures of heart rate and salivary cortisol levels in heroin abstinent patients. Remarkably, these effects were maintained one week after the end of the CBD short-term administration (Hurd et al., 2019). Finally, it is relevant to mention that an exploratory dose ranging study was recently posted in ClinicalTrials.gov to assess the safety, efficacy, and tolerability of APH-1501 (>98.5% CBD, <0.3 THC) for the treatment of opioid dependence. This clinical trial will target opioid-dependent patients completing detoxification in a treatment facility. These will be randomized into 4 treatment groups receiving APH-1501 (400, 600 or 800 mg/m^2^) or placebo over a 30 day period (NCT03813095). Also, another pilot study sponsored by the Johns Hopkins University has been proposed to examine the safety of CBD (Epidiolex) in a human laboratory model of clinically relevant opioid withdrawal. In a residential, randomized and within-subject comparison design, authors will evaluate the effects of placebo and CBD (800 mg) in methadone-maintained patients undergoing spontaneous withdrawal (NCT04238754).

In summary, to date few studies have attempted to demonstrate the efficacy of CBD in opioid addiction. The achievement of promising results lately has motivated further research to evaluate the potential utility of CBD in the management of OUD.

### CBD and Psychostimulants

Stimulant use disorder is defined by the DSM-5 as the continued use of amphetamine-type substances, cocaine, or other stimulants leading to clinically significant impairment or distress, from mild to severe (APA, 2013). The global prevalence of stimulant use has increased over the past decade with a worrying rise in the use of amphetamine-type stimulants and cocaine (United Nations Office on Drugs and Crime, 2019). Amphetamine-type stimulants include substances with a similar chemical structure, such as amphetamine and methamphetamine, and other structurally different but with similar effects, such as methylphenidate. Amphetamine-type stimulants as well as cocaine are highly addictive substances. One of the main concerns is the lack of specific pharmacological tools for the treatment of amphetamine-type or cocaine use disorder. Although psychostimulants have shown some favorable results, high quality clinical trials and meta-analyses are needed to determine their clinical utility (Ronsley et al., 2020). Thus, it is essential to search for new therapeutic approaches. In the last years, many authors evaluated the therapeutic utility of CBD to treat the different phases of dependence to psychostimulants. As reviewed below, published reports focused mainly on evaluating the effects of CBD on the reinforcing and motivational actions of amphetamine, methamphetamine, and cocaine in different animal models (Table 4).

**TABLE 4:** Main findings from clinical and animal studies aimed to evaluate the therapeutic potential of CBD for the treatment of stimulant use disorder.

CBD and psychostimulants					
Treatment	Doses, route of administration, and treatment duration	Study design/model	Subjects, samples, and gender	Main outcomes	References
Amphetamine/methamphetamine					
CBD	5 mg/kg, i.p., 4 days (conditioning pase of CPP) or 1 day (extinction trial)	AMPH-induced CPP	Sprague-dawley rats (M)	= Conditioning score	Parker et al. (2004)
				**↑** CPP extinction	
CBD	10 µg/5 µl, ICV, acute treatment	METH-induced CPP	Wistar rats (M)	↓ METH-induced CPP reinstatement (high priming dose)	Karimi-Haghighi & Haghparast. (2018)
				↓ METH-induced CPP reinstatement (low priming dose in REM sleep deprived rats)	
CBD	10, 20, 40, 80 mg/kg, i.p., repeated treatment (METH-paired conditioning sessions)	METH-induced CPP	Sprague-dawley rats (M)	↓ METH-induced CPP (dose-dependently)	Yang et al. (2020)
CBD	0, 20, 40 and 80 mg/kg, i.p., acute treatment	METH-induced ISA	Male sprague-dawley rats *N* = 32 (M)	↓ motivation to self-administer METH	Hay et al. (2018)
				↓ METH-primed relapse after extinction	
CBD	32 and 160 nmol, ICV, 10 days (abstinence)	Chronic exposure to METH	Wistar rats *N* = 62 (M)	**↑** long-term memory in the NOR test	Razavi et al. (2020)
Cocaine					
CBD	5 mg/kg, i.p., 4 days (conditioning phase of CPP) or 1 day (extinction trial)	Cocaine-induced CPP	Sprague-dawley rats (M)	= Conditioning score	Parker et al. (2004)
				**↑** CPP extinction	
CBD	10 mg/kg, i.p., acute treatment	Cocaine-induced CPP	Wistar rats *N* = 295 (M)	↓ reconsolidation of cocaine-induced CPP	de Carvalho & Takahashi. (2017)
CBD	10 mg/kg, i.p., acute and repeated administration	Cocaine-induced CPP	C57BL/6J mice (M)	↓ preference for the cocaine context	Chesworth & Karl. (2020)
				↓ consolidation of cocaine memory	
				= cocaine-induced CPP	
				= Rate of extinction of cocaine memory	
				= cocaine-primed reinstatement	
CBD	30, 60 mg/kg, i.p., acute treatment	Cocaine-induced CPP	CD1 mice *N* = 120 (M)	↓ cocaine-primed reinstatement	Calpe-Lopez et al. (2020)
				↓ social defeat-induced reinstatement	
CBD	10, 20 mg/kg, i.p., 10 days	Cocaine-induced ISA	CD1 mice (M)	↓ cocaine self-administration and motivation → abolished by hippocampal neurogenesis blockade (temozolomide)	Calpe-Lopez et al. (2020) and Luján et al. (2019)
				= cocaine-induced reinstatement	
CBD	3–20 mg/kg, i.p., repeated administration	Cocaine-induced ISA	Long-evans rats *N* = 75 (M)	↓ cocaine self-administration with low but not high cocaine doses	Galaj et al. (2020)
		Cocaine-induced BSR		↓ cocaine-enhanced BSR	
CBD	15 mg/kg/day, t.d. 7 days	Cocaine-induced ESA	Wistar rats *N* = 52 (M)	↓ context-induced and stress-induced reinstatement	Gonzalez-Cuevas et al. (2018)
CBD + caffeine	20 mg/kg, i.p. Repeated administration	Cocaine-induced locomotor sensitization	Wistar rats (M)	↓ cocaine-induced hyperlocomotion	Prieto et al. (2020)
CBD	10, 20, 40 mg/kg, i.p.Cocaine-induced BSR Acute treatment	Spontaneous cocaine withdrawal	CD1 mice *N* = 100 (M)	↓ anxiety level	Gasparyan et al. (2020)
				↓ hyperactivity	
				↓ withdrawal somatic signs	
CBD	5 mg/kg, i.p. Acute treatment	Cocaine-induced ICSS	Sprague-dawley rats (M)	= reward-facilitating effect of cocaine	Katsidoni et al. (2013)
CBD	5 and 10 mg/kg, i.p. Chronic and acute treatment	Cocaine-induced ISA	Long-evans rats *N* = 40 (M)	= Cocaine self-administration	Mahmud et al. (2017)
				= Cocaine seeking after withdrawal	
CBD	400 or 800 mg/day	Double-blind RCT	Cocaine-dependent individuals *N* = 79 (M/F)	Drug-cue induced craving	ClinicalTrials.gov ID: NCT02559167
				Number of days to relapse (no results posted yet)	

CBD, cannabidiol; AMPH, amphetamine; METH, methamphetamine; CPP, conditioned place preference; ICSS, intracranial self-stimulation; ISA, intravenous self-administration; BSR, brain stimulation reward; NOR, novel object recognition; REM, rapid eye movement; RCT, randomized clinical trial; M, male; F, female; p.o., per os (oral administration); i.p., intraperitoneal injection; ICV, intracerebroventricular; ↑, increase; ↓, decrease; = , no effect.

#### Amphetamine-type Substance Use Disorder

The potential of CBD to modulate amphetamine-induced rewarding properties was first reported in 2004. In this study, the administration of a low dose of CBD (5 mg/kg, i.p.) potentiated the extinction of amphetamine-induced CPP without affecting the learning process of place conditioning (Parker et al., 2004). Years later, another group showed that intracerebroventricular (ICV) injection of CBD (10 µg/5 µl) suppressed the methamphetamine-induced reinstatement in the CPP paradigm, even under stressed conditions (Karimi-Haghighi and Haghparast, 2018). Interestingly, these authors suggested later that the effect of CBD was associated with the normalization of methamphetamine-induced increase of gene expression of cytokines (interleukin‐1β, interleukin‐6, interleukin‐10, and tumor necrosis factor α (TNF‐α)) in the prefrontal cortex (PFC) and HIPP. However, in REM sleep-deprived rats CBD produced opposite effects (Karimi-Haghighi et al., 2020). Recently, CBD-mediated regulation of methamphetamine-induced CPP was further confirmed. Treatment with CBD (10, 20, 40 and 80 mg/kg, i.p.) 1 h prior to the administration of methamphetamine during conditioning sessions significantly and dose dependently attenuated CPP. Importantly, these effects were related with the regulation of the SigmaR1/AKT/GSK-3β/CREB signaling pathway that was up-regulated in the VTA, NAcc, HIPP, and PFC of methamphetamine-treated male Sprague-Dawley rats (Yang et al., 2020). Apart from the effects of CBD on CPP induced by amphetamine and methamphetamine, Hay et al. explored whether CBD modulates the motivation to obtain methamphetamine as well as the relapse into methamphetamine consumption using an intravenous SA paradigm. After a training phase, the administration of CBD (80 mg/kg, i.p.) significantly reduced active lever pressing and consequently the number of methamphetamine infusions, as well as methamphetamine-primed relapse to active lever pressing (Hay et al., 2018).

Chronic exposure to amphetamine-type derivatives could lead to neurodegeneration and neuro-inflammation phenomena with associated cognitive impairments. Therefore, in addition to the interest of modulating rewarding and motivational properties, it is also important to provide a neuroprotective effect to attenuate these alterations. In this sense, a recent report revealed that the ICV administration of CBD during the abstinence period after chronic exposure to methamphetamine (10 days) significantly reverses long-term memory in the novel object recognition test (Razavi et al., 2020). However, more studies are needed to further explore the therapeutic potential of CBD and to elucidate the neurobiological mechanisms involved.

#### Cocaine Use Disorder

One of the first reports suggesting the therapeutic potential of CBD for the modulation of cocaine rewarding properties employed the CPP paradigm. In Sprague-Dawley rats, CBD (5 mg/kg, i.p.) did not change the conditioning score but enhanced CPP extinction (Parker et al., 2004). Also, CBD (10 mg/kg, i.p.) disrupted the reconsolidation of place preference in rats and this effect was present for 2 weeks (de Carvalho and Takahashi, 2017). Very recently, Chesworth and Karl exhaustively explored CBD actions (10 mg/kg, i.p.) on the acquisition, consolidation, reconsolidation, extinction, and drug-primed reinstatement of cocaine (15 mg/kg) in the CPP paradigm. CBD significantly reduced the preference for the cocaine-context and the consolidation of cocaine memory. CBD had no effects on cocaine-induced CPP, the rate of extinction of cocaine memory, or the drug-primed reinstatement (Chesworth and Karl, 2020). However, a recent report of our group demonstrated that CBD (30 and 60 mg/kg, i.p.) significantly reduced cocaine priming- and social defeat-induced reinstatement of CPP (Calpe-Lopez et al., 2020). Likewise, Lujan et al. demonstrated that CBD (10 and 20 mg/kg, i.p.) significantly attenuated cocaine-induced CPP. Furthermore, they employed an intravenous SA paradigm and showed that CBD (20 mg/kg, i.p.) reduced the motivation to self-administer cocaine in a fixed ratio 1 schedule, as well as the breaking point during the progressive ratio stage. Interestingly, CBD effects on cocaine-induced reward and motivation could be related with an increase of CB1R and brain-derived neurotrophic factor (BDNF) expression, MAPK/CREB pathway phosphorylation and neural progenitor proliferation in the HIPP whereas a reduction of GluA1/2 AMPA subunit receptor ratio was found in the striatum of male CD1 mice that underwent cocaine SA (Luján et al., 2018). Also, it is relevant to point out that the effects of CBD on hippocampal neurogenesis plays a pivotal role in the reduction of cocaine SA (Luján et al., 2019). Recently, attenuating effects of CBD on the motivational properties of cocaine were also revealed by Galaj et al.. In this study CBD inhibited cocaine SA maintained by low, but not high, doses of cocaine, and dose-dependently lowered cocaine-enhanced brain-stimulation reward. Importantly, these effects were abolished by the blockade of CB2R, 5HT1a and TRPV1 suggesting their functional implication. Furthermore, *in vivo* microdialysis revealed a CBD-mediated reduction of cocaine-induced increases in extracellular dopamine in the NAcc (Galaj et al., 2020).

In addition to these previous findings, it was also explored whether CBD could be effective to prevent relapse. Gonzalez-Cuevas et al. revealed that the transdermal administration of CBD attenuated context-induced and stress-induced drug-seeking in an intravenous cocaine SA paradigm. Interestingly, CBD-mediated anti-relapsing effects were maintained up to 5 months after the end of the treatment although plasma and brain CBD levels were undetectable at this time (Gonzalez-Cuevas et al., 2018). Furthermore, the effects of CBD on cocaine plus caffeine-induced locomotor sensitization were investigated. Repeated treatment with CBD (20 mg/kg, i.p.) blunted the motor behavioral response induced by a challenge dose of cocaine plus caffeine (Prieto et al., 2020).

Another crucial aspect in the cocaine use disorder is the successful management of cocaine-induced withdrawal syndrome to maintain the abstinence and to prevent relapse. Recently, our group evaluated the role of CBD to regulate behavioral and neurobiological alterations induced by cocaine in a new animal model of spontaneous withdrawal. The results of this study revealed that CBD (10, 20, and 40 mg/kg, i.p.) normalized motor and somatic signs disturbances and completely regulated anxiety-like behaviors induced by spontaneous cocaine withdrawal (progressive increasing doses of cocaine for 12 days, 15–60 mg/kg/day, i.p.). Furthermore, the administration of CBD blocked the increase of dopamine transporter (DAT) and TH gene expressions in the VTA of mice exposed to the cocaine withdrawal (Gasparyan et al., 2020).

On the contrary to the positive findings supporting the therapeutic potential of CBD in the regulation of the reinforcing and motivational actions of cocaine, one study found that CBD (5 mg/kg, i.p.) did not modify the reward-facilitating effect of cocaine in the ICSS paradigm (Katsidoni et al., 2013). Also, another publication showed that CBD (5 and 10 mg/kg, i.p.) did not attenuate the motivation to self-administer cocaine (breaking point) nor the cue-induced cocaine seeking in rats after a withdrawal period (Mahmud et al., 2017). These apparently contradictory results could be related, at least in part, with differences in the experimental design or in the administered doses of cocaine and CBD. However, the available information suggests that CBD could be a useful tool for the treatment of cocaine use disorder although additional studies are warranted.

Finally, a double-blind, randomized and placebo-controlled clinical trial was carried out in 79 patients with cocaine use disorder. The main goal was to evaluate the effects of CBD (400 or 800 mg/day) on cocaine-cue induced craving and the number of days to relapse. Although the results have not yet been published, the performance of this study points out the interest of the therapeutic potential of CBD for cocaine use disorder (NCT02559167).

### CBD and Nicotine

Tobacco use is the cause of over 8 million deaths per year globally, resulting one of the biggest public health threats worldwide (World Health Organization (WHO), 2020). Nicotine is the main addictive substance responsible for cigarette smoking and withdrawal symptoms occurring upon smoking cessation. Nowadays, nicotine replacement therapy together with varenicline, a nicotinic receptor partial agonist, is the most effective smoking cessation drug. However, a significant proportion or smokers still fail to maintain long-term abstinence. Here we reviewed the scarce but recent results pointing out CBD as a candidate to be considered for modulating nicotine-induced reinforcing and withdrawal symptoms.

The first pilot clinical study evaluated the effects of CBD in smokers trying to achieve cessation. Inhaled CBD (400 µg/inhalation) was effective to reduce the number of cigarettes smoked after one week of treatment. Nevertheless, CBD treatment did not attenuate nicotine craving and showed only a slight, non-significant reduction in anxiety after the 7 days treatment (Morgan et al., 2013). A few years later, the administration of a single dose of CBD (800 mg) in non-treatment seeking, dependent, cigarette smokers after overnight abstinence did not improve verbal or spatial working memory, or impulsivity (Hindocha et al., 2018). However, the same group demonstrated that CBD (single 800 mg dose) reduced attentional bias after a period of tobacco abstinence without improving craving or withdrawal (Hindocha et al., 2018). Recently, a preclinical study was conducted to analyze the effects of CBD (10 and 30 mg/kg) in mice exposed to an animal model of pharmacologically precipitated nicotine withdrawal. Interestingly, CBD abolishes memory impairment and microglial reactivity induced by nicotine withdrawal (Saravia et al., 2019).

In summary, although the information on this issue is very limited, it appears that CBD may result an interesting therapeutic alternative for tobacco dishabituation (Table 5). However, further studies should be conducted to improve our knowledge of its usefulness and to increase our understanding of the possible mechanisms involved.

**TABLE 5:** Main findings from clinical and animal studies aimed to evaluate the therapeutic potential of CBD for the treatment of tobacco use disorder.

CBD and nicotine					
Treatment	Doses, route of administration, and treatment duration	Study design/model	Subjects, samples, and gender	Main outcomes	References
Clinical studies					
CBD	400 µg/inhalation solution erosol, inh. 7 days	Double-blind placebo-controlled trial	Smokers *N* = 24 (12 M and 12 F)	↓ number of cigarettes smoked	Morgan et al. (2013)
CBD	800 mg, p.o. Acute treatment	Double-blind placebo-controlled trial	Non-treatment seeking dependent smokers *N* = 30 (15 M and 15 F)	= Verbal or spatial working memory	Hindocha et al. (2018)
				= withdrawal-induced impulsivity	
CBD	800 mg, p.o. Acute treatment	Double-blind placebo-controlled trial	Non-treatment seeking dependent smokers *N* = 30 (16 M and 14 F)	↓ attentional bias	Hindocha et al. (2018)
				↓ pleasantness of cigarette images	
				= Tobacco craving	
				= Withdrawal symptoms	
Animal studies					
CBD	3, 10 and 30 mg/kg, s.c. Repeated treatment	Precipitated nicotine withdrawal	C57BL/6J mice (M)	↑ NOR discrimination index during nicotine withdrawal	Saravia et al. (2019)

CBD, cannabidiol; NOR, novel object recognition; M, male; F, female; inh., inhaled; p.o., per os (oral administration); s.c., subcutaneous injection; ↑, increase; ↓: decrease, = ; no effect.

## Neurobiological Mechanisms Involved in CBD-Mediated Regulation of Addiction

This section is aimed to analyze in an integrated way the mechanisms that could be underlying the “anti-addictive” actions of CBD. For that purpose, the most representative functional brain systems have been selected to dissect which targets and regulatory mechanisms may be modulated by CBD.

### CBD and Dopaminergic System

The scientific community has long accepted the dopaminergic theory of addiction. The hedonic effects of different drugs of abuse are mediated mainly, at least initially, by the release of DA in the mesocorticolimbic system that comprises dopaminergic neurons projecting from the VTA to the NAcc. Released DA in the NAcc acts on high affinity D2 receptors and determines drug rewarding effects (Trifilieff et al., 2013). Also, DA stimulates the low-affinity D1 receptors associated with the consolidation of recent memory engrams (Wise, 2004). However, increased DA levels are not always present after the exposure to a drug of abuse since addiction encompasses a complex functional regulation including the interaction between different neurotransmission systems (Nutt et al., 2015). Despite this, the dopaminergic system plays a central role in addictive disorders.

Little is known about the effects of CBD on the mesolimbic system. One of the first reports revealed that systemically administered CBD had neither excitatory nor inhibitory effects on spontaneously recorded VTA dopaminergic neuronal activity levels (French et al., 1997). In accordance with this finding, systemic injections of CBD alone (10 and 20 mg/kg, i.p.) failed to significantly alter extracellular DA level in the NAcc (Galaj et al., 2020). However, intra-hypothalamic administration of CBD was reported to increase the release of dopamine extracellular levels collected from the NAcc (Murillo-Rodríguez et al., 2011). Due to the antipsychotic actions of CBD, along with the absence of extrapyramidal effects, numerous studies have been conducted to investigate the interaction between CBD and the mesolimbic dopaminergic system employing animal models of schizophrenia. It has been proposed that CBD could act as a partial agonist of D2 receptors (Seeman, 2016) and normalize D3 receptor gene expression in several brain regions (PFC, HIPP, and NAcc) (Stark et al., 2020).

Considering the “anti-addictive” properties of CBD, previously mentioned in this review, it is important to determine how CBD modulates drug-induced alterations in the mesolimbic dopaminergic system. One of the first evidence was published by Renard et al., in an animal model of amphetamine-induced locomotor sensitization. They demonstrated that direct administration of CBD into the shell region of the NAcc completely abolished VTA dopaminergic neuronal activity sensitization induced by amphetamine (Renard et al., 2016). Interestingly, *in vivo* microdialysis studies revealed that systemic administration of CBD (10 and 20 mg/kg, i.p.) dose-dependently attenuated cocaine-induced DA release in the NAcc (Galaj et al., 2020). This effect could be explained by the hypothetical modulation of DA synthesis in the VTA. Indeed, our group has extensively explored the effects of CBD on drug-induced gene expression changes of TH, the rate limiting enzyme for dopamine synthesis in the VTA. In different animal models of ethanol consumption (voluntary consumption, SA, and binge-drinking) the administration of CBD significantly reduced ethanol rewarding and the motivational actions that were associated with a reduction in the gene expression of TH in the VTA (Viudez-Martínez et al., 2018a; Viudez-Martinez et al., 2018b; Viudez-Martínez et al., 2020). Similarly, CBD significantly decreases TH in mice exposed to spontaneous cocaine withdrawal (Gasparyan et al., 2020). Nevertheless, in an animal model of spontaneous cannabinoid withdrawal CBD enhanced TH gene expression in the VTA (Navarrete et al., 2018). These apparent discrepancies could be explained by two main facts. First, DA synthesis and release vary throughout the different phases of the addictive process, depending on whether consumption or withdrawal stages are present. Second, DA release in the NAcc depends on the mechanism of action of each drug of abuse. Accordingly, cannabis, unlike alcohol and psychostimulants would present a minimal effect that could account for these opposite regulations (Nutt et al., 2015).

Thus, available evidence suggests that CBD may functionally regulate the activity of the mesolimbic DA system and counteract the effects of dysregulated dopaminergic transmission induced by drugs such as amphetamine, cocaine, alcohol, or cannabis. These findings could be related, at least in part, to the reduction of the reinforcing and motivational effects of these drugs, as well as to the regulation of the withdrawal syndrome. Nevertheless, more studies are needed to precisely explore CBD-mediated regulation of dopaminergic mechanisms involved in drug addiction.

### CBD and Opioidergic System

The endogenous opioid system is closely involved in the regulation of addictive behaviors. Opioid peptides do not directly affect dopaminergic neurons function in the VTA but inhibit gamma-aminobutyric acid (GABAergic) interneurons that innervate VTA dopaminergic neurons in the mesolimbic system (Johnson and North, 1992). The activation of MOR in the VTA through its endogenous ligands, β-endorphin and enkephalin, disinhibits the inhibition produced by the GABAergic interneurons and increases DA release in the NAcc whereas the selective blockade of these receptors significantly decreases basal DA release (Spanagel et al., 1992). Some drugs of abuse (e.g., alcohol, cannabis) stimulate the release of endogenous opioids leading to a MOR-mediated increase of DA release in the NAcc (Tanda and Di Chiara, 1998).

The interaction between CBD and opioidergic system components has been barely explored. A few studies evaluated changes in the main targets of the opioidergic system after CBD administration. The first reference was published in 2006 by Kathmann et al. They described the CBD-mediated allosteric modulation of mu- and delta-opioid receptor by means of kinetic binding studies with ^3^H-DAMGO (D-Ala^2^, NMePhe^4^, Gly-ol) in the cerebral cortex membrane of male Wistar rats. These effects only occur at very high concentrations and cannot be expected to contribute to the *in vivo* action (Kathmann et al., 2006). Recently, our group analyzed MOR gene expression changes after CBD administration in animal models of alcohol addiction. Interestingly, CBD-induced reduction of voluntary ethanol consumption, ethanol SA and binge-drinking was associated with a down-regulation of MOR in the NAcc (Viudez-Martínez et al., 2018a; Viudez-Martinez et al., 2018b; Viudez-Martínez et al., 2020). Similarly, the administration of CBD normalized increased MOR gene expression in the NAcc in mice exposed to an animal model of spontaneous cannabinoid withdrawal (Navarrete et al., 2018). Therefore, independently of the experimental paradigm employed, the phase of addiction assessed and the drug, the effect of CBD was in the same direction. Thus, it is possible to speculate that CBD negatively modulates MOR; however, more studies should be carried out to further explore the specific interaction between CBD and MOR receptors, as well as with other components of the opioidergic system.

In summary, available evidence suggests that CBD-induced modulation of drug reinforcing and motivational properties could be mediated, at least in part, by the functional regulation of the opioidergic system. However, it remains to elucidate the precise mechanisms involved.

### CBD and Endogenous Cannabinoid System

ECS is a ubiquitous lipid signaling system distributed throughout the organism that participates in multiple intracellular signaling pathways (Piomelli, 2003; Zou and Kumar, 2018). ECS regulates several physiological functions and mediates the crosstalk between different neurotransmitter systems, therefore, representing a key player in the control of behavioral responses (Katona and Freund, 2012; Atkinson and Abbott, 2018). CB1R and CB2R, endogenous ligands or endocannabinoids (AEA and 2-arachidonoylglycerol (2-AG)), and their synthesizing (N-acylphosphatidylethanolamine specific phospholipase D (NAPE-PLD) and diacylglycerol lipases (DAGL-α and DAGL-β)) and degrading (FAAH and monoacylglycerol lipase (MAGL)) enzymes are the main components of the ECS, present in the central and peripheral nervous system (Mackie, 2005; Katona and Freund, 2012). As recently and extensively reviewed by our group and other authors, ECS is critically involved in the neurobiological substrate underlying drug addiction. Importantly, the functional localization of cannabinoid receptors in the mesocorticolimbic circuit participating in the modulation of the synthesis and release of dopamine is widely accepted (Maldonado et al., 2006; Parsons and Hurd, 2015; Sloan et al., 2017; Trigo and Le Foll, 2017; Manzanares et al., 2018).

Numerous studies were carried out to elucidate the interactive mechanisms between CBD and ECS components. One of the mechanisms is the inhibition of AEA hydrolysis and reuptake by blocking its catabolic enzyme (FAAH) and the corresponding membrane transporter, respectively (Bisogno et al., 2001; Laprairie et al., 2015). Regarding the interaction with CB1R, CBD was first thought to be an antagonist (Thomas et al., 2007; Pertwee, 2008), but recent results suggested that CBD could act also as a non-competitive negative allosteric modulator of CB1R (Laprairie et al., 2015; Tham et al., 2019). Interestingly, a statistical meta-analysis of all present information describing direct effects of CBD at cannabinoid receptors concluded that there is no direct CBD–CB1R interaction that may account for the reported changes in endocannabinoid signaling (McPartland et al., 2015).

There is also controversy about the pharmacological effect of CBD on CB2R. It was proposed that CBD could act as a partial agonist (Tham et al., 2019), inverse agonist or even as an antagonist (Thomas et al., 2007). A recent report suggested that CBD might act as an allosteric modulator (Martinez-Pinilla et al., 2017). Finally, CBD presents recognized antagonistic properties on GPR55 receptor (Ryberg et al., 2007; Sharir and Abood, 2010; Ibeas Bih et al., 2015).

The findings published by our group demonstrated that CBD down-regulates the gene expression of CB1R and GPR55 whereas up-regulates CB2R in the NAcc of C57BL/6J mice exposed to models of cannabinoid withdrawal (Navarrete et al., 2018) and alcohol addiction (Viudez-Martínez et al., 2018a; Viudez-Martínez et al., 2020). These effects may be related, at least in part, with CBD-mediated improvement of withdrawal symptoms and the reduction of alcohol consumption, motivation, and relapse. Similarly, Ren et al. showed a reduction of CB1R gene and protein levels in the NAcc core and shell subregions of rats exposed to a cue-induced heroin seeking procedure. Interestingly, these authors suggested that the effects of CBD on CB1R expression would present a mesolimbic specificity (Ren et al., 2009). Furthermore, CBD increased CB1R protein expression in the HIPP of mice exposed to a cocaine SA paradigm (Luján et al., 2018). On the other hand, the antagonism of CB2R by the administration of AM630 completely blocked the reduction of cocaine SA by CBD, suggesting its critical involvement in CBD-mediated effects (Galaj et al., 2020).

Taken together, it is possible to argue that ECS components play a pivotal role in the actions of CBD on withdrawal-related, reinforcement, motivation or relapse induced by alcohol, cocaine, or heroin. Thus, a greater effort is essential to further characterize the mechanisms involving the ECS that underlies potential therapeutic effects of CBD in drug addiction.

### CBD and Serotonergic System

The serotonergic system has a pivotal role in the modulation of motivational and reinforcement processes and is involved in the regulation of the rewarding effects of certain drugs of abuse. Mesolimbic dopaminergic neurons are critically regulated by serotonergic projections from the medial and dorsal raphe nuclei entailing an inhibitory control (Di Giovanni et al., 2010; Müller and Homberg, 2015). There are a high number of serotonergic receptors subtypes with different functional profiles, suggesting the complexity of serotonin-mediated regulation of drug reward. Among these, 5HT1a receptors stand out due to the large number of reports supporting its crucial role in drug addiction (Pinto et al., 2002; Risinger and Boyce, 2002; Müller et al., 2007; Kelaï et al., 2008; You et al., 2016). Importantly, CBD is known to act as a positive allosteric modulator at 5HT1a receptors (Russo et al., 2005; Campos and Guimarães, 2008) and this mechanism is closely involved in its anxiolytic and antidepressant actions (Fogaça et al., 2014; Linge et al., 2016; Sartim et al., 2016). Furthermore, the modulation of dopamine release in the NAcc by CBD was described and it appears to occur through a mechanism involving the activation of 5HT1a receptors (Norris et al., 2016).

To investigate the role of 5HT1a receptors in the CBD-mediated regulation of drug reward, our group analyzed the gene expression in the dorsal raphe (DR) of C57BL/6J mice that underwent an ethanol SA paradigm. Interestingly, the reduction in ethanol consumption and motivation induced by CBD was accompanied by a reduction of 5HT1a gene expression (Viudez-Martínez et al., 2018b). Pharmacological approaches employing the 5HT1a antagonist WAY-100635 confirmed the involvement of this receptor in the effects of CBD on drug-induced reward. First, intra-dorsal raphe injection of WAY-100635 abolished the CBD-mediated inhibition of the reward-facilitating effect of morphine measured in the ICSS paradigm (Katsidoni et al., 2013). Second, the administration of the selective 5HT1a antagonist completely blocked CBD plus naltrexone effects on ethanol SA (Viudez-Martínez et al., 2018b). Third, blockade of 5HT1a receptors attenuated CBD-mediated reduction of cocaine SA (Galaj et al., 2020). Therefore, all these results suggest that the effects of CBD on drug reward and motivation are mediated, at least in part, by 5HT1a receptors. It is tempting to hypothesize that the activation of these receptors by CBD in brain areas of the mesocorticolimbic circuit may play a critical role. A great effort is necessary to further elucidate and understand the interaction of CBD with the serotonergic system and its involvement in drug addiction.

### CBD and Glutamatergic System

Glutamate (Glu) is the main excitatory neurotransmitter of the central nervous system. Glutamatergic synaptic plasticity in the mesocorticolimbic dopaminergic circuit is a key neuronal process in appetitive learning and significantly contributes to the development and maintenance of drug addiction (Yamaguchi et al., 2011; van Huijstee and Mansvelder, 2014). Drugs of abuse trigger critical adaptive changes in the reward system by inducing widespread modifications of glutamatergic synapses. The NAcc receives glutamatergic projections from the VTA (Yamaguchi et al., 2011) and other regions involved in the addictive process such as PFC, amygdala, and HIPP (Koob and Volkow, 2010; Floresco, 2015; Heinsbroek et al., 2020). The acquisition of drug reward associations depends on the convergence of dopaminergic and glutamatergic signaling in the NAcc (Neuhofer et al., 2019). Thus, glutamatergic neurotransmission plays an important role in the functional regulation of relevant brain structures involved in the neurocircuitry of drug addiction.

Few studies evaluated the effects of CBD on the different components of glutamatergic signaling in drug reward in animal models of addiction. The administration of CBD inhibited cue-induced heroin seeking in an intravenous SA paradigm and increased AMPA GluR1 protein levels in the NAcc core and shell subregions, achieving a normalization effect. However, mGluR5 protein levels were not modified by CBD (Ren et al., 2009). Also, CBD significantly reduced AMPA GluR1/2 protein levels in the striatum of mice self-administering cocaine (Luján et al., 2018). Finally, in an animal model of cocaine-induced intoxication, the administration of CBD reduced cocaine-induced seizures and this effect was associated with the activation of the mTor pathway with a subsequent significant reduction on Glu release in hippocampal synaptosomes (Gobira et al., 2015).

Therefore, although the available information is very limited, it is reasonable to suggest that CBD-mediated regulation of glutamatergic neurotransmission plays a crucial role in the modulation not only of drug reward but also of drug-induced neuroadaptive changes. However, more studies are needed to confirm this notion and to explore the effects of CBD in other targets of the glutamatergic system.

### CBD and Hippocampal Neurogenesis

In recent years, the major role of hippocampal neurogenesis in the addictive process has become increasingly established (Mandyam and Koob, 2012; Chambers, 2013; Deroche-Gamonet et al., 2019). A number of reports suggests that psychoactive substances with addictive potential modify neurogenesis in the adult HIPP (Castilla-Ortega et al., 2016). The subventricular zone (SVZ) and the subgranular layer of the hippocampal dentate gyrus (DG) are the brain regions where adult neurogenesis occurs. Drugs such as psychomotor stimulants, opioids or alcohol significantly impair several aspects of adult neurogenesis including the rate of progenitor proliferation, the survival of newly generated cells and the maturation and acquisition of cellular phenotype (García-Fuster et al., 2010; Taffe et al., 2010). These alterations may affect several drug-related psychological processes such as learning, memory and mood regulation (Canales, 2007).

One of the first reports showing the pro-neurogenic effect of CBD was published by Wolf et al.. The treatment with CBD (6 weeks) enhanced adult neurogenesis; however, this effect was not present in mice lacking CB1R suggesting the critical role of these receptors in the CBD-mediated actions on hippocampal neurogenesis (Wolf et al., 2010). The anxiolytic actions of CBD in mice exposed to chronic unpredictable stress were closely associated with the pro-neurogenic effect of CBD. The authors suggested that this phenomenon depends on the facilitation of the endocannabinoid-mediated signaling and subsequent cannabinoid receptors activation (Campos et al., 2013; Fogaça et al., 2018). Likewise, repeated administration of CBD at low doses (3 mg/kg, i.p.) increased cell proliferation and neurogenesis in the DG and SVZ (Schiavon et al., 2016). Interestingly, a recent critical review covers the potential therapeutic implications of the pro-neurogenic effects of CBD for the treatment of distinct psychiatric disorders, including drug addiction (Lujan and Valverde, 2020).

Recent advances focused on the study of the molecular basis that underlies the neurogenesis promoting actions of CBD in relation with the regulation or drug reward. Lujan et al. described that CBD increased neural progenitor proliferation in the HIPP of cocaine self-administering animals. They explored the activation of MAPK pathway and its downstream pathways that regulate the expression of the transcriptional (CREB) and neurotrophic (BDNF) factors, responsible for the levels of neuronal hippocampal proliferation. Interestingly, the administration of CBD up-regulated ERK1/2 and CREB phosphorylation, as well as BDNF expression in the HIPP of mice that underwent cocaine SA. Furthermore, the number of BrdU/NeuN stained cells in the HIPP was significantly higher in CBD-treated animals (Luján et al., 2018). To further confirm the involvement of adult hippocampal neurogenesis in the CBD-mediated actions on cocaine reward, Lujan et al. carried out an elegant study administering temozolomide (25 mg/kg/day), a chemotherapy drug that blocks hippocampal neurogenesis. The results clearly demonstrated that in absence of the neurogenesis processes CBD does not modulate cocaine consumption and motivation (Luján et al., 2019). Thus, additional studies are warranted to further explore the therapeutic potential of CBD in addictive disorders regarding its pro-neurogenic as well as neuroprotective properties.

## Concluding Remarks

The present review shows the current state of the art about the potential interest of CBD as a new pharmacological avenue for SUD. According to the findings from preclinical and clinical studies, CBD alone or in combination with commonly employed treatment strategies in drug addiction may configure a potential therapeutic option for improving the dishabituation process of addicted patients.

The great interest in the promising profile of CBD for the management of SUD was revealed by the significant number of clinical studies published or currently underway. One of the most representative examples is CUD for which numerous clinical trials evaluated the effects of CBD, mostly in combination with THC, on withdrawal symptoms, craving, and cannabis use. The information with CBD alone is still insufficient due to the small number of patients in the studies that were carried out to date. Additional clinical trials with more patients and longer treatment periods are warranted to further explore the efficacy and safety of CBD for the treatment of CUD. Interestingly, the results reported by our group in an animal model of spontaneous cannabinoid withdrawal support the implementation of randomized controlled trials (RCT) using only CBD. In addition, variables like motivation, reinforcement, withdrawal, relapse, and retention in treatment should be considered for a global overview during treatment for CUD. Smoking is another SUD in which clinical studies were predominantly conducted to evaluate CBD actions. Nevertheless, more information is required to accurately assess the therapeutic role that CBD could have in smoking cessation. Importantly, one of the main current limitations is the low oral bioavailability of CBD that requires the joint effort to develop new oral formulations to ensure adequate plasma levels and consequently reduce pharmacokinetic variability (Millar et al., 2018; Izgelov et al., 2020; Perucca and Bialer, 2020).

On the other hand, the role of CBD in alcohol, opioid and psychostimulant use disorders lies mainly in the studies carried out with different animal models, which in turn motivated the performance of several ongoing clinical trials. The findings included in this review suggest that CBD may reduce the consumption, motivation or relapse of alcohol, opioids (i.e., heroin, morphine) and psychostimulants (amphetamine, methamphetamine, and cocaine), as well as the withdrawal-related signs of morphine and cocaine. The clinical trials recently launched will provide relevant information to know the outcome of the translational approach to patients suffering from these addictive disorders. In addition, it is important to highlight the protective actions derived from CBD treatment not only to attenuate drug-induced damages in the CNS, but also in peripheral tissues such as alcohol-induced liver steatosis or cirrhosis.

**FIGURE 1:**
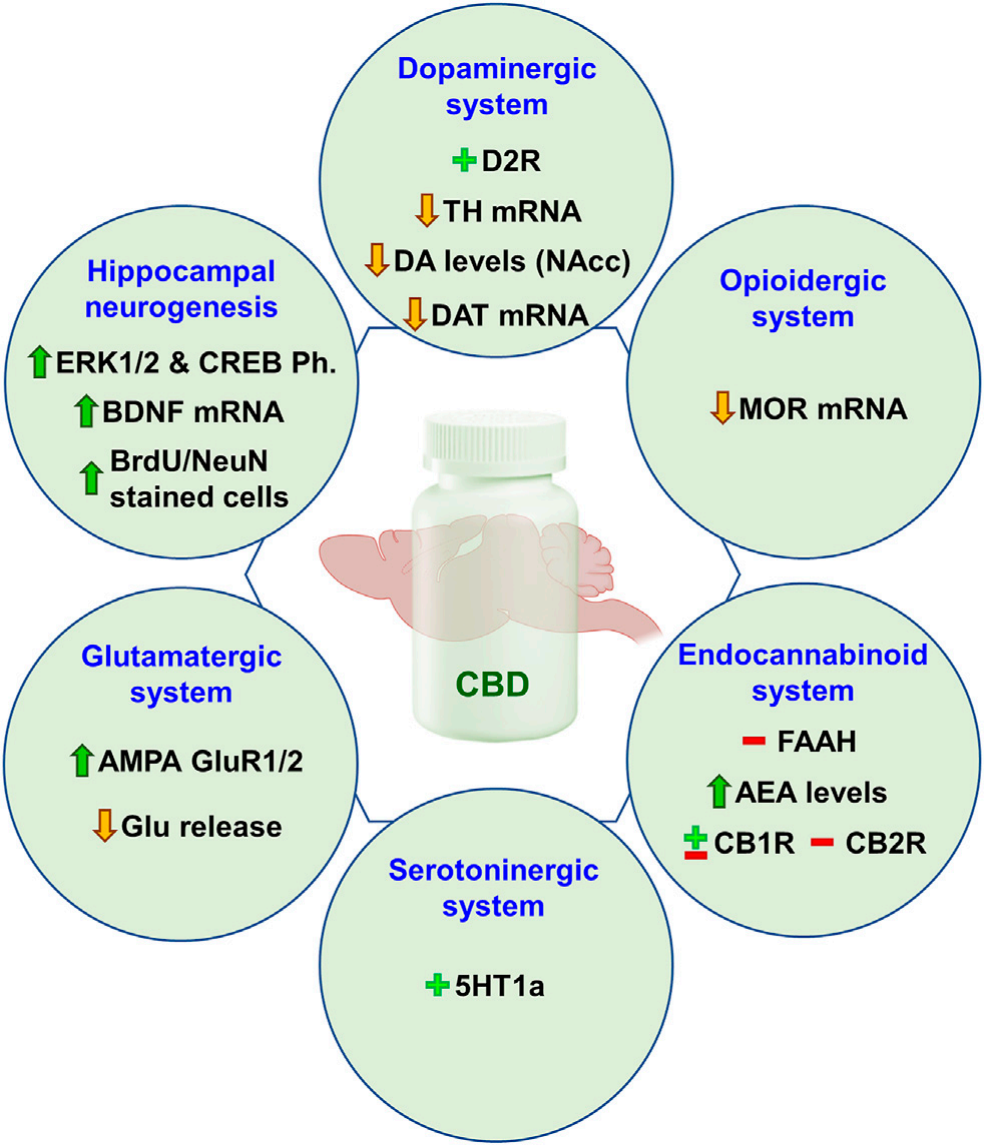
Main preclinical findings regarding the neurobiological mechanisms underlying the “anti-addictive” potential of CBD in relation with dopaminergic, opioidergic, endocannabinoid, serotonergic, and glutamatergic systems, as well as hippocampal neurogenesis. D2r, dopamine receptor 2; TH, tyrosine hydroxylase; DA, dopamine; NAcc, nucleus accumbens; MOR, mu-opioid receptor; FAAH, fatty acid amide hydrolase; AEA, anandamide; CB1R, cannabinoid receptor 1; CB2R, cannabinoid receptor 2; 5HT1a, serotonin receptor 1a; AMPA GluR1/2, α-amino-3-hydroxy-5-methyl-4-isoxazolepropionic acid glutamate receptor 1/2; Glu, glutamate; CREB, cAMP response element-binding protein; Ph., phosphorylation; BDNF, brain-derived neurotrophic factor.

A fundamental aspect to optimize the therapeutic potential of CBD in the treatment of SUD is to improve our knowledge about the mechanisms that are involved in its actions. For that reason, the present review dedicates a special section to the interaction between CBD and distinct neurotransmission or functional regulation systems (Figure 1). Taking into account all the information that has been collected in this respect, the following ideas can be highlighted: 1) CBD can modulate dopaminergic neurotransmission in the mesolimbic circuit through the direct regulation of dopamine synthesis, release or effects on dopamine receptors, or by indirect mechanisms as the modulation of MOR; 2) the ECS plays a pivotal role in CBD-mediated effects on drug reward, involving the regulation of endocannabinoid signaling through the alteration of AEA levels and CB1R or CB2R function; 3) 5HT1a receptors are critically involved in the effects of CBD on drug addiction; and 4) hippocampal neurogenesis appears to be essential for the regulation of cocaine consumption and motivation by CBD.

In summary, we have ahead of us an exciting race to discover how CBD could contribute to the area of drug addiction from a therapeutic point of view. More preclinical and clinical studies are necessary to further evaluate the role of CBD as a new therapeutic intervention for SUD. In this regard, it is relevant to emphasize that according to the multiple pharmacological profile of CBD accounting for the anxiolytic, antidepressant or antipsychotic properties, comorbid clinical entities such as anxiety, depression or psychotic disorders could be also successfully managed. Importantly, taking into consideration the sex biological differences in terms of brain function and connectivity and its relationship with distinct vulnerability to develop a substance use disorder (Becker et al., 2017), it could be argued that CBD may display differential effects depending on sex (Viudez-Martínez et al., 2020), an aspect that needs to be further explored. The clinical studies that are currently underway will provide relevant information to improve our knowledge about the efficacy and safety of CBD for the treatment of SUD.

## Abbreviations

2-AG, 2-arachidonoylglycerol; 3H-DAMGO, D-Ala^2^, NMePhe^4^, Gly ol; 5HT1a, serotonin 1a receptor; 5HTT, serotonin transporter; AEA, anandamide; Amy, amygdala; AMPA, α-amino-3-hydroxy-5-methyl-4-isoxazolepropionic acid receptor; AUD, alcohol use disorder; BDNF, brain-derived neurotrophic factor; BPN, buprenorphine; CB1R, cannabinoid receptor 1; CB2R, cannabinoid receptor 2CBD, cannabidiol; CBT, cognitive behavioral therapy; CNS, central nervous system; CPA, conditioned place aversion; CPP, conditioned place preference; CREB, cAMP response element-binding protein; CUD, cannabis use disorder; DAGL, diacylglycerol; DALYs, disability-adjusted life years; DAT, dopamine transporter; DG, dentate gyrus; DR, dorsal raphe; DSM-5, diagnostic and statistical manual of mental disorders, 5th version; ECS, endocannabinoid system; EMA, European Medicines Agency; EtOH, Ethanol; FAAH, fatty acid amide hydrolase; FDA, Food and Drug Administration; FR1, fixed-ratio 1; FR3, fixed-ratio 3; FMRI, functional Magnetic Resonance Imaging; GPCRs, Gq protein-coupled receptors; Glu, glutamate; HIPP, hippocampus; ICD-11, International Classification of Diseases; ICSS, intracranial self-stimulation; ICV, intracerebroventricular; MAGL, monoacylglycerol lipase; MAPK, mitogen-activated protein kinase; MET, motivational enhancement therapy; MOr, mu-opioid receptor; NAcc, nucleus accumbens; NAPE-PLD, N-acylphosphatidylethanolamine specific phospholipase D; NMDAr, N-methyl-d-aspartate receptors; NTX, naltrexone; OUD, opioid use disorder; PFC, prefrontal cortex; PTSD, post-traumatic stress disorder; RCT, randomized controlled trial; RT-PCR, real time polymerase chain reaction; SA, self-administration; SUD, substance use disorder; SVZ, subventricular zone; THC, delta-9-tetrahydrocannabinol; THC-COOH, 11-nor-9-carboxy-δ-9-tetrahydrocannabinol; TH, tyrosine hydroxylase; TNF-α, tumor necrosis factor α; VEC, voluntary ethanol consumption; VTA, ventral tegmental area; YLDs, years lived with disability.
